# The Prevalence of Micronutrient Deficiencies and Inadequacies in the Middle East and Approaches to Interventions

**DOI:** 10.3390/nu9030229

**Published:** 2017-03-03

**Authors:** Nahla Hwalla, Ayesha Salem Al Dhaheri, Hadia Radwan, Hanan Abdullah Alfawaz, Mona A. Fouda, Nasser Mohammed Al-Daghri, Sahar Zaghloul, Jeffrey B. Blumberg

**Affiliations:** 1 Faculty of Agricultural and Food Sciences, American University of Beirut, Beirut 1107 2020, Lebanon; nahla@aub.edu.lb; 2 Nutrition and Health Department, College of Food and Agriculture, United Arab Emirates University, Al Ain, UAE; ayesha_aldhaheri@uaeu.ac.ae; 3 College of Health Sciences, Clinical Nutrition and Dietetics Department, University of Sharjah, Sharjah, UAE; Hradwan@sharjah.ac.ae; 4 Department of Food Science and Nutrition, College of Food Science and Agriculture, King Saud University, Riyadh, Saudi Arabia; halfawaz@ksu.edu.sa; 5 Department of Medicine, Division of Endocrinology, College of medicine and KSUMC, King Saud University, Riyadh, Saudi Arabia; monafoudaneel@yahoo.com; 6 Prince Mutaib Chair for Biomarkers of Osteoporosis, Biochemistry Department, College of Science, King Saud University, Riyadh, Saudi Arabia; ndaghri@ksu.edu.sa; 7 Department of Nutrition Requirements and Growth, National Nutrition Institute, Cairo, Egypt; zaghloulsahar@gmail.com; 8 Friedman School of Nutrition Science and Policy, Jean Mayer USDA Human Nutrition Research Center on Aging, Tufts University, Boston, MA 20111, USA

**Keywords:** Middle East, micronutrient, deficiency, inadequacy, dietary supplementation, food fortification

## Abstract

Micronutrient deficiencies and inadequacies constitute a global health issue, particularly among countries in the Middle East. The objective of this review is to identify micronutrient deficits in the Middle East and to consider current and new approaches to address this problem. Based on the availability of more recent data, this review is primarily focused on countries that are in advanced nutrition transition. Prominent deficits in folate, iron, and vitamin D are noted among children/adolescents, women of childbearing age, pregnant women, and the elderly. Reports indicate that food fortification in the region is sporadic and ineffective, and the use of dietary supplements is low. Nutrition monitoring in the region is limited, and gaps in relevant information present challenges for implementing new policies and approaches to address the problem. Government-sponsored initiatives are necessary to assess current dietary intakes/patterns, support nutrition education, and to reduce food insecurity, especially among vulnerable population groups. Public–private partnerships should be considered in targeting micronutrient fortification programs and supplementation recommendations as approaches to help alleviate the burden of micronutrient deficiencies and inadequacies in the Middle East.

## 1. Introduction

Micronutrient deficiencies have long been a major healthcare problem in the Middle East. However, over the past three decades, the region has been subject to substantial changes in the demographic, economic, political, and social environment that impact the challenges associated with diet, nutrition, and health. Many countries in the Middle East are undergoing a nutrition transition where undernutrition coexists with non-communicable diseases associated with other forms of malnutrition (including overweight and obesity). The World Health Organization (WHO) has divided this region into overlapping country clusters with regard to nutrition stages and dominant nutrition problems, including major risk factors and underlying causes, program interventions, and gaps in response to these problems [1]. Countries in advanced nutrition transition have a high prevalence of overweight and obesity and a moderate prevalence of undernutrition and micronutrient deficiencies in some population subgroups; these countries include Bahrain, Iran, the Kingdom of Saudi Arabia (KSA), Kuwait, Oman, Qatar, and the United Arab Emirates (UAE). Countries in early nutrition transition (e.g., Egypt, Jordan, Lebanon, Morocco, and Palestine) are typically characterized by a moderate prevalence of overweight and obesity, moderate levels of undernutrition in specific population groups, and widespread micronutrient deficiencies/inadequacies. Countries with significant undernutrition (e.g., Djibouti, Iraq, Pakistan, and Yemen) have a high prevalence of acute and chronic malnutrition, widespread micronutrient deficiencies, and emerging overweight, obesity, and malnutrition of affluence in certain socioeconomic groups. Countries in complex emergency situations present with severe child and maternal undernutrition and widespread micronutrient deficiencies; these countries include Afghanistan, Libya, Somalia, Sudan, and Syria.

With regard to micronutrient intakes/status, the WHO identifies particularly deficient and/or inadequate intakes or status of calcium, iodine, iron, and zinc as well as vitamin A, vitamin D, and folate as commonly reported by many countries in the Middle East region, particularly in children and women of childbearing age [2]. Over one-third of the population in the region is iron deficient or anemic, the majority of whom are women. Surveys have estimated that 13.2 million preschool children have a serum retinol status <0.70 µmol/L, with 0.8 million afflicted by night blindness. About one-third of the population are at risk of iodine deficiency disorders, including cognitive and functional development in early childhood [2].

In addition to documenting these micronutrient deficiencies and/or inadequacies, the WHO has also stressed the growing public healthcare challenge presented by non-communicable diseases, accounting for 47% of morbidity and 52% of total mortality [2]. Much of this total is derived from conditions associated with poor nutrition; for example, in those aged ≥20 years, 11% were diabetic, 26% were hypertensive, 50% were dyslipidemic, and 65% were overweight or obese. Among people aged ≥15 years from Middle East countries, those from Bahrain, Egypt, Jordan, KSA, Kuwait, and the UAE had the highest prevalence of overweight and obesity at 74%–86% in women and 69%–77% in men.

Dietary guidelines have been promoted in the Middle East recommending diets rich in fruits, vegetables, and whole grains, and limited in added sugar, solid fats, and sodium [3,4,5]. Although these recommendations for well-balanced and varied diets represents the fundamental approach to meeting recommended micronutrient allowances, the percentage of those achieving this goal is small. In addition to insufficient micronutrient intakes due to poor diet, inadequacies and deficiencies can arise from conditions of impaired absorption, chronic disease(s), and/or drug-induced inadequacies [6,7]. Beyond the inherent risk for deficiency states, the consequence of long-term inadequate intake of vitamins and minerals can be associated with decrements in cellular function, physiological performance, and/or resilience, as well as an increased risk for chronic disease [7,8,9].

Although improvements in nutrition in some Middle East countries are taking place as a result economic growth and associated health sector development, the burden of disease associated with inadequate dietary intake remains the immediate cause of undernutrition, with the situation worsening in certain areas as a result of the nutrition transition. This review focuses on the prevalence and impact of micronutrient deficiencies and inadequacies in select Middle Eastern countries and discusses approaches to diet, fortification, and supplementation that may contribute to solving this problem.

## 2. Rationale and Approach

Pfizer Consumer Health in the Middle East sponsored a workshop of eight invited nutrition experts from Egypt (SZ), Lebanon (NH), KSA (HAAF, MFN, and MMA-D), the UAE (ASAD and HR), and the United States (JBB) to discuss the prevalence of micronutrient deficiencies and inadequacies in the region and to consider current interventions and new approaches to address these problems. A pre-meeting survey was conducted to gather insights and views on local practices and challenges regarding dietary guidance and the use of dietary supplements. The workshop was convened on 25–26 March 2016, in Dubai, UAE. For use prior to and post meeting, an online folder hosting information pertaining to the meeting and key references was created and shared with all participants.

After review of these materials, and based on the abundance, quality, and reliability of the available data, evaluation of the scientific literature was principally directed to the Gulf countries (Bahrain, KSA, Kuwait, Oman, Qatar, and UAE), Levant states (Jordan and Lebanon), and Egypt, all countries noted as being in advanced nutrition transition [1]. This narrative review is largely limited to results obtained from Egypt, KSA, Lebanon, and UAE, due principally to the relatively better quality of data and their publication within the last 15 years. Further, due to absence of large, nationally representative surveys and randomized controlled trials conducted across the Middle East, data from cross-sectional studies, case-control studies, and small targeted surveys are reviewed and summarized here to provide an overview of micronutrient intakes and status and highlight the need to put nutrition at the forefront of regional healthcare planning and policy.

### Literature Review Approach

This literature review was conducted using Medline, Google Scholar, and reports of the Food and Agriculture Organization of the United Nations (FAO) and WHO. Data were primarily reviewed from articles published from 2000 to 2016. Only four articles published between 1989 and 2000 (three from 1994 to 1998; one from 1989) were included due to their important and unique information. The common search terms used included:

- Nutrition, undernutrition, malnutrition, nutrition transition, nutrition status, public health, overweight, obesity, obese, cardiovascular, non-communicable diseases, food consumption
- Prevalence, incidence, burden
- Micronutrient deficiency, vitamins, minerals
- Iron deficiency, folate, folate deficiency, anemia, iron deficiency anemia, iron supplementation
- Vitamin D deficiency, vitamin D insufficiency, vitamin D supplementation
- Vitamin A deficiency, night blindness, vitamin A supplementation
- Dietary supplement, multivitamin mineral supplement, benefit, safety, upper limit, lower limit, clinical, non-clinical
- Fortification, flour, iodine, salt, sodium, vitamins, folic acid, vitamin B12, riboflavin, thiamine, vitamin A, minerals, iron, zinc, calcium
- Healthcare cost, health economics

The search was also conducted to be country-, region-, and age-specific. The search terms listed above were used in combination with the following terms:

- Middle East, Egypt, Saudi Arabia, Saudi, KSA, UAE, United Arab Emirates, Kuwait, Bahrain, Qatar, Oman, Lebanon, Jordan, Gulf, GCC, Gulf countries, Arab countries
- Global, world-wide, international, USA, US, Europe, Asia (used only for Section 5, Section 6 and Section 7 below)
- Children, preschool, adolescents, adults, men, women, elderly, older adults

## 3. Prevalence of Micronutrient Deficiencies in the Middle East

### 3.1. Vitamin D

Despite abundant, year-long sunshine in the Middle East, vitamin D deficiency and inadequacy is prevalent due, in large part, to traditional clothing covering most of the body and the lack of foods rich in, or fortified with, vitamin D.

When reviewing available data on vitamin D deficiency from Middle Eastern countries, comparisons were difficult due to the diverse cut-off points used to define deficiency and the varied assays used to measure it. Internationally, and according to the map developed by the International Osteoporosis Foundation [10], a 25-hydroxyvitamin D (25(OH)D) serum level of <25 nmol/L is generally acknowledged as indicating vitamin D “deficiency”, a serum level of 25–49 nmol/L as “insufficient”, 50–74 nmol/L as “inadequate” and >75 nmol/L as “sufficient”. Reported means on vitamin D are not discussed here because they do not provide a measure of the number of people who are deficient in vitamin D or the prevalence of vitamin D deficiency in the region.

Using the aforementioned classification, findings on vitamin D deficiency from the four countries (Egypt, KSA, Lebanon, and UAE) were not abundant in literature, but ranged between 14% and 50%. Available data reported the lowest deficiency among Saudi children (14.2%) [11] and elderly males in Egypt (19.3%) [12], whereas the highest deficiency was reported among Saudi adolescents and women of childbearing age (50%) [11].

Insufficiency levels were reported more abundantly across almost all population groups in the four countries and were found to be higher than deficiency levels, ranging from 11.5% in Egyptian children [13] to 86.4% in Saudi pregnant women [14].

#### 3.1.1. Children and Adolescents

The highest prevalence of vitamin D deficiency and insufficiency among children and adolescents in the Middle East was reported from Lebanon, the UAE, and the KSA, with a range of 45%–62% [11,15,16,17]. Lowest levels were found among Saudi and Egyptian children (14.2% and 11.5%, respectively) [11,13].

In Lebanon, an investigation by El-Hajj Fuleihan et al. of vitamin D status in 164 boys and 182 girls aged 10–16 years across two seasons found that >50% presented with vitamin D insufficiency (serum 25(OH)D < 50 nmol/L), with the prevalence greater in boys than in girls, and highest in winter (65%) and lowest in summer (40%) [15]. Similarly, in a retrospective study of 349 Lebanese patients ≤18 years of age, 45%–62% were found to have vitamin D insufficiency (serum 25(OH)D < 50 nmol/L) in 2007–2008 [18]. Poor vitamin D status was found to improve in 179 Lebanese girls, aged 10–17 years, who were supplemented with weekly doses of 1400 or 14,000 IU vitamin D for one year, with associated benefits observed in lean mass, bone area, and total hip bone mineral content, especially in premenarchal girls [13].

In Egypt, a cross-sectional study by Abu Shady et al. of 200 school children, aged 9–11 years, reported vitamin D inadequacy (serum 25(OH)D 50–74.8 nmol/L) and insufficiency (serum 25(OH)D < 50 nmol/L) in 15% and 11.5% of the cohort, respectively [19]. Obesity, low physical activity, limited sun exposure, and a mother with low educational level were significant predictors of inadequacy. Female sex, a mother with low educational level, and low milk intake were significant predictors of insufficiency.

In a UAE cohort of mothers and their rachitic children, a positive correlation was observed with both groups presenting with vitamin D deficiency (serum 25(OH)D < 25 nmol/L) [16]. A cross-sectional study of 315 healthy adolescents in the UAE by Muhairi et al. found that 45.4% had vitamin D insufficiency (serum 25(OH)D ≤ 50 nmol/L) [20]. Adolescent girls were also more likely to have a serum 25(OH)D level of ≤37.5 nmol/L than boys (28% vs. 10%, respectively); interestingly, serum 25(OH)D was inversely correlated with weekly consumption of fast food and body mass index (BMI).

In the KSA, a case-control study by Al-Mustafa et al. of 61 rachitic infants (mean age, 14.8 months) and 58 controls (mean age, 16.5 months) found that 75% of patients were deficient in vitamin D (serum 25(OH)D < 20 nmol/L), compared with 25% of control children, with breastfeeding and limited sun exposure more common in the cases [21]. In a study of 34 Saudi adolescents, rickets was 59% and attributed to vitamin D deficiency, 12% to low calcium intake, and 29% to genetic causes [22]. Similarly, 42 Saudi children and adolescents diagnosed with rickets or osteomalacia had limited sun exposure and low calcium intake associated with poor serum concentrations of 25(OH)D [17]. A study in the KSA of 113 boys and 147 girls by Al-Daghri et al. found 22% of boys and 49.5% of girls with vitamin D deficiency (serum 25(OH)D < 25 nmol/L), with fresh milk consumption, especially in girls, moderately associated with mean serum 25(OH)D [11]. A retrospective study in the KSA of 10,709 patients, found that vitamin D deficiency (25(OH)D < 25 nmol/L) was more prevalent among adolescents compared with other age groups (49.2% vs. 30.9%, respectively) [23]. This study involved 2178 children aged 12 years, of whom 12% and 49.1% of boys and 18.2% and 47.6% of girls were categorized as having vitamin D deficiency (25(OH)D < 25 nmol/L) and insufficiency/inadequacy (25(OH)D 25–74 nmol/L) [23].

#### 3.1.2. Women and Pregnancy

No prevalence was reported on vitamin D deficiency among pregnant women in the Middle East; however, high insufficiency levels were found in the KSA (86.4%) [14] and Egypt (40%) [23].

For example, Al-Faris found that, among 160 pregnant Saudi women in their first-trimester, 50% and 43.8% had insufficient and inadequate vitamin D at <50 nmol/L and 50–74 nmol/L serum 25(OH)D, respectively [14]. In the KSA, vitamin D deficiency and insufficiency are increasing among women post-delivery, with 59% at <25 nmol/L 25(OH)D) in 1984 and 86.4% at <50 nmol/L 25(OH)D in 2016 [24,25].

A study by El Rifai et al. of 135 pregnant Egyptian women at ≥37 weeks’ gestation (immediately before delivery) reported vitamin D insufficiency (serum 25(OH)D < 50 nmol/L) and inadequacy (serum 25(OH)D 50–80 nmol/L) in 40% and 28.9% of patients, respectively [26]. In another study, 54% of 50 pregnant women and 72.6% of 51 lactating women were found to have insufficient vitamin D (serum 25(OH)D < 50 nmol/L) [27]. Neonatal serum 25(OH)D was consistently found to correlate with maternal serum status in studies reported from Egypt and the KSA [25,26,28].

#### 3.1.3. Women of Childbearing Age

The prevalence of vitamin D deficiency and insufficiency is high among women of childbearing age in the Middle East, ranging from 24% to 72% across the four countries [11,17,26,28,29].

In an observational study of 60,979 subjects from the UAE, Haq et al. found vitamin D deficiency, insufficiency, and inadequacy in 26.4%, 35%, and 21.6% of 35,066 women with serum 25(OH)D status of ≤24.9, 25–49.9, and 50–74.99 nmol/L, respectively [30]. Al Anouti et al. examined 138 Emirati women aged 20.8 ± 4.0 years and found the level of mean serum 25(OH)D to be higher in summer than in winter, suggesting that seasonal variation plays a role in vitamin D status [31]. In a retrospective study of vitamin D status among 82 women residing in the UAE, Sridhar et al. found that 51.8% were deficient, 42.8% were insufficient, 25.4% were inadequate, and 30.8% had sufficient levels, categorized as a serum 25(OH)D of <25, 25–50, 52.5–75, and >75 nmol/L, respectively [32].

Al Attia et al. reported serum 25(OH)D < 75 nmol/L in 90.5% of 255 adult women (mean age 44 years) from the UAE [33]. Interestingly, traditional clothing was not found to significantly affect vitamin D status, with observed rates of serum 25(OH)D < 75 nmol/L equally high among women who were fully veiled, veiled with hands and face exposed, and those who were unveiled (i.e., wearing Western clothing). By contrast, a study published over a decade earlier including 75 women reported that traditional clothing adversely impacts vitamin D status, with deficiency and insufficiency serum 25(OH)D levels recorded in UAE nationals (21.5 nmol/L) and other non-Gulf Arabs (31.5 nmol/L), compared with sufficient levels found in European women (160.8 nmol/L) [29].

In Lebanon, Gannage-Yared et al. studied 318 female hospital workers with a mean age of 40.4 ± 11 years and reported vitamin D deficiency (serum 25(OH)D < 23.5 nmol/L) in 25% and insufficiency (serum 25(OH)D 23.75–53.75 nmol/L) in 50% of women, with no significant correlations with BMI or age [34].

Abdelkarem et al. investigated 147 female Saudi students, aged 18–25 years, and found that 55.3% presented with sufficient serum 25(OH)D status (>75 nmol/L), while 27.3% and 17.4% were classified as inadequate (50–72.5 nmol/L) and insufficient (<50 nmol/L), respectively [35]. Compared with students of normal weight and those who are overweight, a higher percentage of obese students were found to be vitamin D insufficient (25(OH)D levels of <50 nmol/L in 0.9% of normal weight students, 0.8% in overweight students vs. 54.6% in obese students) [35]. Among another group of Saudi women, Al-Daghri et al. found that half (*n* = 71) were vitamin D deficient (serum 25(OH)D < 25 nmol/L) and insufficient (serum 25(OH)D 25–50 nmol/L) [11]. They also found that overall dairy consumption in these women was moderately associated with serum 25(OH)D concentration. In a retrospective study, Hussain et al. observed 10,709 Saudi patients and reported that 51.2% and 44.1% of adult women (aged 19–60 years) had vitamin D deficiency and insufficiency/inadequacy at <25 and 25–75 nmol/L, respectively, with a higher prevalence of deficiency reported among women than men [23].

In Egypt, 96 apparently healthy female college students, mean age 20.8 years, were recruited by Fawzi et al. to investigate the prevalence of vitamin D deficiency and whether vitamin D status was related to dress style [36]. Similar to the findings in the UAE reported by Al Attia et al. [33], no difference was noted by Fawzi et al. in the prevalence of vitamin D insufficiency (25(OH)D < 50 nmol/L) or inadequacy (25(OH)D 50–72.5 nmol/L) in Western (30.6% insufficient; 44.4% inadequate), Hegab (34.9% insufficient; 46.5% inadequate) or Nekab (29.4% insufficient; 52.9% inadequate) dress styles [36]. Botros et al. studied 208 women with a mean age of 31.5 years, and reported vitamin D insufficiency in 72% (serum 25(OH)D < 50 nmol/L) [27]. In contrast to the report by Fawzi et al. [36], in the study by Botros et al. vitamin D levels were significantly higher in non-veiled females (57.5 nmol/L) than veiled females (41.8 nmol/L) [27]. El Sagheer et al. examined 100 women of childbearing age (20–40 years) and found them to be vitamin D insufficient [37]; through multiple variable linear regression analysis, independent factors associated with vitamin D insufficiency were a lack of sun exposure, lack of vitamin D supplementation, and the presence of pseudofracture.

#### 3.1.4. Men

Data on micronutrient deficiencies in men residing in the Middle East appears limited and mainly focuses on vitamin D, with the highest deficiency and insufficiency levels reported in Lebanon at 27% and 54%, respectively [34], as well as in the UAE and KSA at ~40% [11,28,38].

In a cross-sectional national survey, Tuffaha et al. examined 10,735 Saudi patients aged ≥15 years and found that 40.6% of men had serum 25(OH)D < 70 nmol/L [39]. A retrospective study by Hussain et al. reported similar data from 10,709 Saudi patients, with 40.2% and 53.5% of men (*n* = 3363) reported as having vitamin D deficiency and insufficiency/inadequacy at 25(OH)D < 25 and 25–75 nmol/L, respectively [23]. Based on data from three smaller cross-sectional studies in the KSA of 1084 men, the prevalence of vitamin D insufficiency, defined as 25(OH)D ≤ 50 nmol/L, ranged between 10% and 92% in those aged >20 years [38,40,41]. Two of these studies indicate that, in the KSA, the prevalence of vitamin D inadequacy at 50–72.5 nmol/L has increased from 18%–25% in 2009 to 80%–88% in 2011 [38,42]. These studies also noted that the rate of vitamin D inadequacy was similar in men aged 25–35 years and those aged ≥50 years. Al-Daghri et al. examined vitamin D status in the Eastern region of the KSA and found that 64% of 249 men were vitamin D deficient (<25 nmol/L) and insufficient (25–50 nmol/L) [11]; in this cohort, a higher percentage of men were vitamin D deficient or insufficient than women (*n* = 316) despite >65% of all participants having adequate exposure to sunlight and >90% reporting adequate intake of dairy products.

In Lebanon, in a cohort of 392 subjects including 74 men, Gannage-Yared et al. found vitamin D deficiency (serum 25(OH)D < 23.5 nmol/L) and insufficiency (serum 25(OH)D 23.75–53.75 nmol/L) in 27% and 54% of men, respectively, with no significant differences between genders [34]. Vitamin D status was found to be influenced by season, weekly hours of sun exposure, and fish consumption. Similarly, in a study of 316 Lebanese adults (99 men and 217 women), Gannage-Yared et al. reported that 72.8% of patients were deficient in vitamin D (25(OH)D < 30 nmol/L), with deficiency less common in men than in women (48.5% and 83.9%, respectively) and consistent with their average daily vitamin D intake (127.5 ± 79.7 vs. 87.9 ± 57.9 IU, respectively) [43].

In an observational study of 60,979 residents in the UAE, Haq et al. reported 18.4%, 39.9%, and 23.6% of men (*n* = 25,913) with deficient, insufficient, and inadequate vitamin D status at ≤24.9, 25–49.9, and 50–74.0 nmol/L, respectively, despite year-long sunshine [30]. In a study of 70 UAE men aged 21.0 ± 4.6 years, Al Anouti et al. found serum 25(OH)D to be higher in summer than in winter [31]. In a retrospective study of vitamin D status among 243 multiethnic men residing in UAE, Sridhar et al. found that 48.2% were deficient, 57.2% were insufficient, 74.6% were inadequate, and 69.2% had sufficient serum 25(OH)D at <25, 25–50, 52.5–75, and >75 nmol/L, respectively [32].

In Egypt, El Tayeb et al. randomly tested 59 men, aged 40–59 years, and found 62.7% had sufficient serum 25(OH)D ≥ 75 nmol/L, with 13.6% and 23.7% having inadequate (50–72.5 nmol/L) and insufficient (<50 nmol/L) status, respectively [44]. Those men in the latter two categories had significantly less sun exposure and lower consumption of dairy products.

#### 3.1.5. Older Adults

Most studies on vitamin D deficiency and insufficiency among the elderly in the Middle East have been conducted on women [26,45,46,47,48], a few have included both genders [11,15,49,50], and only one has been conducted solely on men [12]. Low prevalence of vitamin D deficiency has been reported among the elderly in Egypt at 19.3% [12] and KSA at 23.1% [23]; however, insufficiency levels were found to be higher, reaching 41% among the elderly in Lebanon [15] and 77.2% among those in Egypt [26]. Vitamin D insufficiency among older adults in the region has been attributed to increasing age, low vitamin D intake, female gender, low exposure to sunlight, and veiling (although the results on veiling are mixed) [45].

In older postmenopausal women from Lebanon, 84.9% had serum 25(OH)D levels <75 nmol/L [51]. In a retrospective study of 2896 Lebanese subjects ≥65 years of age, Hoteit et al. reported vitamin D insufficiency in 41% of all elderly subjects using criteria of <50 nmol/L 25(OH)D [15].

Several observational studies from the KSA have reported a prevalence of vitamin D insufficiency (serum 25(OH)D < 50 nmol/L) in the range of 19%–85% in postmenopausal women [42,46,47,48]. For example, in a cohort of 671 postmenopausal women, Ardawi et al. found 34.1% of women presented with vitamin D insufficiency (serum 25(OH)D < 50 nmol/L) and secondary parathyroidism evident in 24.6% of women, with lower vitamin D status and higher intact parathyroid hormone in the upper quintiles of BMI and waist-to-hip ratio [48]. In apparently healthy postmenopausal women with normal bone mineral density, Alissa et al. found vitamin D insufficiency (serum 25(OH)D < 50 nmol/L) in all subjects, with serum vitamin D reported as lower than that of women with osteopenia, and attributable to poor intakes of calcium and vitamin D [52]. In a retrospective study by Hussain et al. of 1304 Saudi patients >60 years of age (embedded within a larger cohort), 21.9% and 61.1% of men and 26.6% and 59.6% of women had vitamin D deficiency and insufficiency/inadequacy defined as serum 25(OH)D of <25 and 25–75 nmol/L, respectively [23].

In a cross-sectional study of 404 Egyptian women, Botros et al. found the prevalence of vitamin D insufficiency to be 39.5% and 77.2% in 38 older subjects (mean 58 ± 4.2 years) and 57 geriatric patients (mean 76 ± 6.7 years), respectively [27]. In a cross-sectional study of vitamin D status in 57 male and 43 female patients aged ≥60 years, Mahmoud et al. found that 16% were deficient (serum 25(OH)D ≤ 30 nmol/L) and 52% were insufficient/inadequate (serum 25(OH)D 30–75 nmol/L) [49]. A higher rate of vitamin D deficiency was observed in women than men (20.9% vs. 12.3%, respectively); however, a higher rate of vitamin D insufficiency/inadequacy was observed in than in women men (66.7% vs. 32.6%, respectively). Similarly, a study by El Araby et al. of 88 randomly selected hospitalized elderly men (≥60 years of age) found that 19.3% and 79.5% of patients were vitamin D deficient (serum 25(OH)D < 25 nmol/L) and insufficient/inadequate (serum 25(OH)D 25–72.5 nmol/L), respectively [50]. By contrast, in a study of 178 community dwelling older adults ≥60 years, representative of the rural areas in Egypt, Aly et al. found no cases of vitamin D insufficiency, although the prevalence of inadequacy (serum 25(OH)D 50–72.5 nmol/L) was observed in 26% of those studied, with higher rates recorded in men (37.9%) than in women (15.3%) [12].

**Figure 1:**
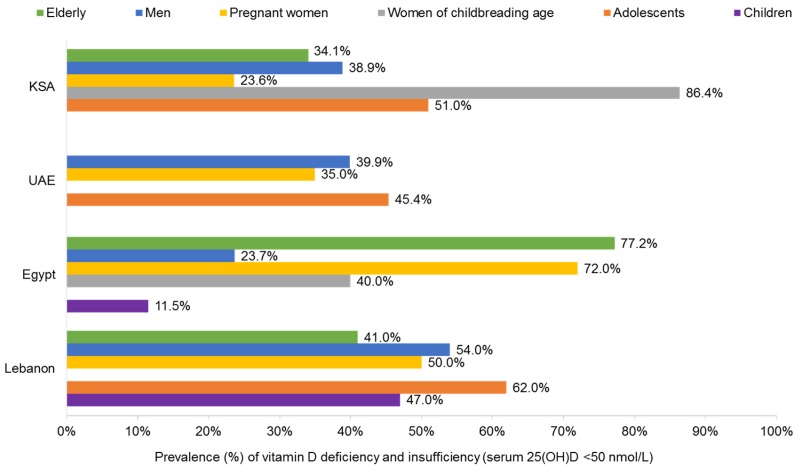
Prevalence of vitamin D deficiency and insufficiency (serum 25(OH)D < 50 nmol/L) in the Middle East by age and gender (Sources: KSA [11,24,40,48]; UAE [20,30]; Lebanon [18,34]; Egypt [19,26,27,44]).

Although clearly an important health issue in the Middle East, the limited number and scope of published studies and the variable definitions of deficient, insufficient, inadequate, and sufficient 25(OH)D status makes comparison of this problem with other regions difficult. Nonetheless, it is worth noting that, in the systematic review of international results by Fairfield and Fletcher, among 75,647 postmenopausal women from 25 countries on five continents, 24% had insufficient vitamin D status (serum 25(OH)D 25–50 nmol/L), as indicated by elevated serum parathyroid hormone [53]. Similarly, Fairfield and Fletcher cite studies from the United States revealing that 50% of postmenopausal women admitted with hip fractures were vitamin D deficient (serum 25(OH)D ≤ 30 nmol/L) and 57% of medical in-patients were vitamin D deficient (serum 25(OH)D < 37.5 nmol/L), with 22% being severely deficient (serum 25(OH)D < 20 nmol/L). A summary of the prevalence of vitamin D insufficiency in selected Middle Eastern countries is provided in Figure 1.

### 3.2. Iron, Folate, and Anemia

In investigating iron status in children, adolescents, women, and the elderly in the Middle East, most studies have used decreased serum hemoglobin to indicate anemia, decreased serum/plasma ferritin with normal hemoglobin levels to indicate iron deficiency or depleted iron stores, and decreased plasma ferritin together with decreased hemoglobin levels to indicate iron deficiency anemia (IDA).

Measuring hemoglobin levels alone reflects the general prevalence of anemia, a multifactorial disease arising from many vitamin and mineral deficiencies. In women of childbearing age, insufficient consumption of folate, vitamin B12 and vitamin A, repeated pregnancies, menorrhagia, postpartum hemorrhage, gastric ulcers, hemorrhoids, intake of aspirin/non-steroidal and anti-inflammatory drugs, and consumption of pure vegetarian diets are all factors that contribute to anemia [54]. Moreover, measuring ferritin levels alone could result in misdiagnosis, because ferritin concentrations increase as a result of parasitic infections, worm infestations, and prevalent inflammatory conditions, making interpretation based on serum ferritin alone (i.e., iron deficiency) unreliable [55].

In this report, the prevalence of anemia in the Middle East was reported among all age groups except men, with the highest rates documented among Egyptian children, adolescents, and women, reaching up to 47% [56]. The lowest prevalence of anemia was found among Saudi school-aged children and the elderly at approximately 12% [57,58].

#### 3.2.1. Children and Adolescents

Anemia is the most prevalent nutritional disorder among children in the Middle East and North Africa region [59]. The prevalence of anemia in children and adolescents ranges from 11.6% in Saudi school-aged children [60] to 39.6% in Egyptian preschool children [61]. As classified by the WHO, the prevalence of anemia, as a public health issue globally, describes a mild problem at 5%–19.9%, a moderate problem at 20%–39.9%, and a severe problem at ≥40%; most Arab Middle East countries fall within the category of moderate to severe deficiency [57,62].

In a cross-sectional study on 11,800 school children conducted in the UAE by Musaiger et al. in 1994–1995, the total prevalence of anemia among six year olds was 31% (Hb < 12 g/dL) with Emirati boys more likely to develop anemia than girls and expatriate children [63]. In a cross-sectional, home-based survey in the UAE of 496 children, Miller et al. found that 36.1% were anemic and 9.9% were iron deficient [64]. They also reported age as a significant independent predictor of both iron depletion and IDA; current pregnancy in the mother was an additional predictor of IDA [59].

In KSA children, Babiker et al. undertook a historical cross-sectional study, including 333 children, and found that the prevalence of IDA (transferrin saturation < 10% and serum ferritin <12 µg/L) by age was 3.3%, 9.3%, 12.7%, and 14.5% in children aged 5–6, 7–8, 9–10, and 12–15 months of age, respectively [65]. In a cross-sectional study of 500 Saudi infants aged 6–24 months attending a well-baby clinic, Al Hawsawi et al. reported that 49% had IDA (Hb < 11 g/dL, serum ferritin <10 µg/L) [66]. In a study of Saudi school children, Abou-Zeid et al. reported 11.6% to be anemic, with a declining rate of anemia from ages 6 to 14 years [60]. It is worth noting that many children in this study were underweight (14.2%), stunted (12.2%), obese (9.8%), or suffered from wasting (13.8%) with anemia.

In the KSA, the prevalence of anemia in young people aged 15–21 years ranged from 16% to 34% with Hb < 12 g/dL [67,68]. Anemia was reported by Abalkhail et al. in 20.5% of 800 Saudi students at 10.3%, 20.6%, and 24.6% for those aged 9–11, 12–14, and 15–21 years, respectively [67]; at the highest risk for developing anemia were those 12 years of age, of low social class, and menstruating girls. Similarly, in cross-sectional, community-based survey in the KSA of 203 males and 192 females aged 13–18 years, Alquaiz et al. revealed that 16.7% of males and 34.2% of females had anemia with Hb < 12 g/dL [68]. This study showed significant associations between anemia and female gender, family history of IDA, overweight, no or little intake of fresh juices, and living in an apartment or a small house.

In a cross-sectional study of 300 Egyptian infants, 6–24 months old, Elalfy et al. reported that 66% had anemia (Hb < 11 g/dL), of which 43% had IDA [56]. The principal risk factors associated with IDA were age 6–18 months, male sex, higher birth order, consumption of cow’s milk, predominant breastfeeding >6 months, and/or low iron intake. In another cross-sectional study of 300 infants and children, 0.5–12 years, conducted a pediatric outpatient clinic in Egypt, Al Ghwass et al. found that 64% of patients had IDA, with a distribution of 20%, 41.7%, and 2.3% for mild, moderate, and severe cases, respectively [69]. In a community-based survey of 397 Egyptian children, aged 0.5–15 years, Barakat et al. reported that 18% had anemia (Hb < 11 g/dL) and 32.5% had manifestations of iron deficiency [70]. Of those classified as anemic, 56.5% of pre-school children were <2 years and 11% of school children were 6–9 years [70]. A study of 4526 households from 11 governorates in Egypt, recorded anemia in 35.7% of 6816 adolescents (Hb < 12 g/dL), 35.3% of 2620 schoolchildren (Hb < 11.5 g/dL), and 39.6% of 4376 preschool children (Hb < 11 g/dL) [61]. Iron deficiency was observed in 47.4% of 5020 adolescents and 38.2% of 1853 schoolchildren both with serum ferritin < 15 µg/L, and 38.2% of 2345 preschool children with serum ferritin < 12 µg/L. Mousa et al. examined the prevalence of iron deficiency and IDA in 912 adolescent girls residing in five different villages from Upper Egypt, wherein 39.9% were anemic (Hb < 12 g/dL), 30.2% had IDA (Hb < 12 g/dL, serum ferritin < 15 ng/mL), and 11.4% were iron deficient without anemia (serum ferritin < 15 ng/mL) [71].

Austin et al. examined the trends in anemia (Hb < 11 g/dL) from the Egyptian Demographic and Health Survey (EDHS) conducted between 2000 and 2005, and revealed a prevalence of 37%–52% among Egyptian children aged 12–36 months [59]. This rise in the prevalence of anemia was attributed to shifting food consumption patterns and increases in childhood diarrhea. According to the EDHS 2014 report, IDA appears to be declining [72]. Nonetheless, more than one in four young children in Egypt suffer from some degree of anemia, with 10% moderately anemic and the remainder classified as mildly anemic. Rural children were more likely to be anemic than urban children (29% and 23%, respectively). Children 9–12 months were found to have the highest prevalence of anemia at 49.2%, whereas those between 48 months and 59 months had the lowest prevalence at 16.1%. Moreover, the proportion of children <5 years with any anemia in the 2014 EDHS at 27.2% was similar to the level reported in the 2000 EDHS at 30.3%, but considerably lower than the level reported in the 2005 EDHS at 48.5%. Girls aged 5–19 years were slightly more likely than boys in this age group to be anemic (21% and 18%, respectively), with the highest prevalence reported in 12–14 year olds at 25% and the lowest in 10–11 year olds at 14%. Among boys, anemia was highest in the 15–19 age group at 22%, and lowest in the 10–11 age group at 10% [73].

#### 3.2.2. Women and Pregnancy

In an earlier survey of 6539 pregnant women from the KSA, Mahfouz et al. found the prevalence of anemia at 31.9% [74]. In a cross-sectional, community-based study in Egypt, Rezk et al. reported a prevalence of IDA at 51.3% in pregnant women (microcytic anemia with Hb < 10.5 g/dL at the second trimester with serum ferritin < 25 ng/dL) [75]. Using self-reported data from 270 pregnant Egyptian women, Ibrahim et al. reported that 75% were mildly anemic (Hb 9–11 g/dL) and 23.2% were moderately anemic (Hb 7–9 g/dL) [76]. Of those diagnosed with anemia, only 35.7% compiled with recommendations to supplement with folic acid plus iron.

According to the 2000, 2005, and 2014 EDHS reports, a marked reduction in IDA among pregnant women, consistent with the increased use of iron supplements, was observed [72,77,78]. The estimated prevalence of anemia in pregnant women also declined from 45% in 2000 and 34.2% in 2005, to 20.6% in 2014, whereas iron supplement use increased from 39.4% in 2000 and 56.5% in 2005, to 66.6% in 2014.

#### 3.2.3. Women of Childbearing Age

The prevalence of anemia among women of childbearing age differs between Middle Eastern countries; lowest rates were documented in Lebanon at 16% [79] followed by UAE at 26.7% [80], whereas the highest rates were reported in the KSA and Egypt at 40%–47.2% [56,81].

In the UAE, Sultan et al. reported the prevalence of anemia at 26.7% (Hb < 12 g/dL) among 258 female college students, with 88.4%, 7.2%, and 2.3% presenting with mild, moderate, and severe (Hb < 7 g/dL) cases [82].

In a survey of 969 women of childbearing age in the KSA, Alquaiz et al. reported a 40% prevalence of anemia (Hb < 12 g/dL) [83]. An earlier study by Al-Quaiz et al. of 87 anemic and 203 non-anemic women in the KSA found the risk of IDA diagnosis to increase 2–4 fold due to the low frequency of eating meat, vegetables, and/or drinking juices with vitamin C [84]. The risk of IDA increased 3–6 fold in women with a menstrual period duration of >8 days, history of clots, or heavy bleeding.

A cross-sectional survey by Al Khatib et al. of 470 women (aged 15–45 years) visiting government health centers in Lebanon, reported that 16% were anemic (Hb < 12 g/dL), 27.2% were iron deficient (ferritin < 15 µg/L) and 7.7% were diagnosed with IDA [85].

A study of 4526 households from 11 governorates in Egypt, found that 47.2% of 4526 mothers were anemic (Hb < 12 g/dL) and 49.6% of 3037 mothers were iron deficient (serum ferritin < 15 µg/L) [61]. According to the 2014 EDHS report, 25% of women, 15–45 years, were mildly anemic [72]. The rate of anemia was highest among women living in rural Upper Egypt and lowest among women from the three Frontier Governorates at (31% vs. 20%, respectively).

#### 3.2.4. Older Adults

**Figure 2:**
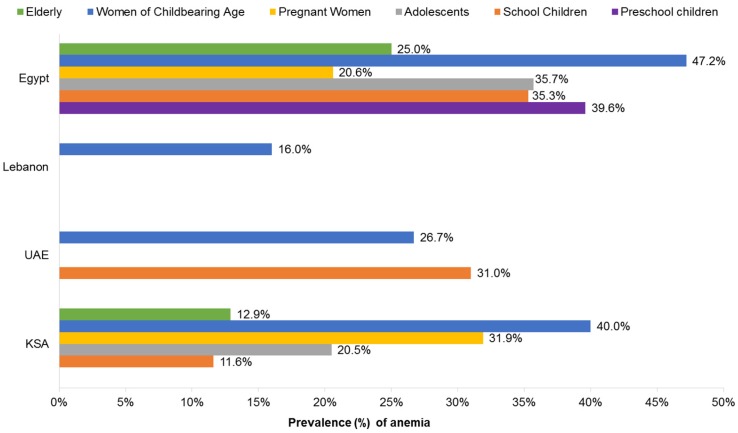
Prevalence of anemia in the Middle East by age and gender. (Sources: KSA [60,67,83,86,88]; UAE [63,82]; Lebanon [85]; Egypt [61,72,89]).

In a KSA survey of 150 adults >60 years of age, Alsaeed et al. found the overall prevalence of anemia to be 12.9%, with a higher proportion seen in women than men (18% vs. 5%, respectively) [86]. A national survey conducted in Egypt, including 4707 adults aged ≥65 years, reported the prevalence of anemia at 25% (Hb < 12 g/dL) [87]. A summary of prevalence data on anemia in the Middle East is provided in Figure 2.

#### 3.2.5. Neural Tube Defects and Folate Deficiency

Multiple micronutrient deficiencies often co-exist in women during pregnancy, and may lead to adverse maternal outcomes [74]. Pregnancy and lactation increase the requirement for folate but typical dietary intakes in the Middle East do not provide the necessary amount. Several observational studies conducted in the KSA, assessing the incidence of neural tube defects (NTD), report an incidence of 1.05–1.90/1000 live births [90,91,92], highlighting the lack of dietary folate and inadequate access to folate-fortified foods or dietary supplements. A similar incidence of NTD has been reported in other Middle Eastern countries, ranging from 1.0 to 3.3/1000 live births, despite reported folic acid fortification in these countries [58,79,80,81,93,94,95,96,97].

In Egypt, a survey conducted by Tawfik et al. reported the prevalence of folate deficiency (<10 nmol/L) at 14.7% among 579 mothers [89]. A cross-sectional survey by Al Khatib et al. of 470 women (aged 15–45 years) visiting government health centers in Lebanon, found folate deficiency (<6.6 ng/mL) at 25.1% [85]. Data on the prevalence of folate deficiency among children and adolescents and older adults in the Middle East are not available.

### 3.3. Vitamin A

Based on a pooled analysis of population-based surveys in the Middle East, Stevens et al. reported vitamin A deficiency in children aged 6–59 months, with a prevalence of 10% [98]. A similar prevalence of vitamin A deficiency was reported in surveys conducted in the KSA, each concluding that this deficit in vitamin A intake does not represent a major public health problem, although functional outcomes were not assessed [99,100,101]. Eight years after the implementation of a vitamin A supplementation program in Egypt in 1999, Tawfik et al. assessed vitamin A status in a national survey and found that 6% of preschool children and 3.7% of their mothers had serum retinol level <20 µg/dL, suggesting the effectiveness of the program [102]. In the UAE, Qazaq et al. evaluated 198 pregnant women for vitamin A deficiency and found 6% presenting with plasma retinol < 20 µg/dL [103]; hematological indices tended to be lower in these women, although the differences were not statistically significant. The WHO global database on vitamin A deficiency in the Middle East reports that the prevalence of night blindness and vitamin A deficiency (plasma retinol < 0.7 µmol/L) ranges from 0.1% to 0.6% and 1.0% to 12% in pre-school children and from 3.7% to 5.1% and 3.0% to 10.2% in pregnant women, respectively (Table 1) [104]. These data from WHO report that vitamin A deficiency is not a public health problem in Bahrain, Kuwait, Qatar, and the UAE.

## 4. Deficient, Inadequate, and Sufficient Micronutrient Intakes

Deficiency syndromes associated with very low intake of specific vitamins and essential minerals are well established and can be treated by administering the specific micronutrient at the proper dose for the necessary duration before irreversible pathology occurs. Less specific, generalized complaints of fatigue, lethargy, and/or pain in the absence of a frank deficiency syndrome or other underlying disease are often associated with inadequate intakes or stores of vitamins and minerals, although subsequent assessment by healthcare professionals of dietary intake or nutritional status is not often conducted. Nonetheless, it is worth noting a dose–response relationship exists between stages of sufficient, inadequate, marginal, and deficient intakes (relative to recommended dietary allowances or accepted ranges of oral intake) and bioclinical markers without apparent functional impact, subclinical biomarkers of functional impairment, clinical signs and symptoms, and deficiency syndrome with irreversible pathology, respectively [106,107]. The early stages in this progression may result from poor diet, malabsorption conditions, and/or altered metabolism due to aging, disease or drug therapy. Challenges associated with assessing these stages and their risk for deficiency syndromes or the risk for acute or chronic diseases include: (a) clinical and anatomical defects may be late manifestations of micronutrient deficiency; (b) assessment of nutrient status/stores is imperfect, particularly for micronutrients with homeostatic mechanisms maintaining circulating concentrations and/or for which storage pools are not in close equilibrium with circulating levels; and (c) early biomarkers predicting deficiency syndromes or associations with other disease conditions are limited [108]. Complicating this schema is that the duration of progression from one stage to another is dependent upon a multiplicity of factors including age, health status, and baseline storage depots.

**Figure 3:**
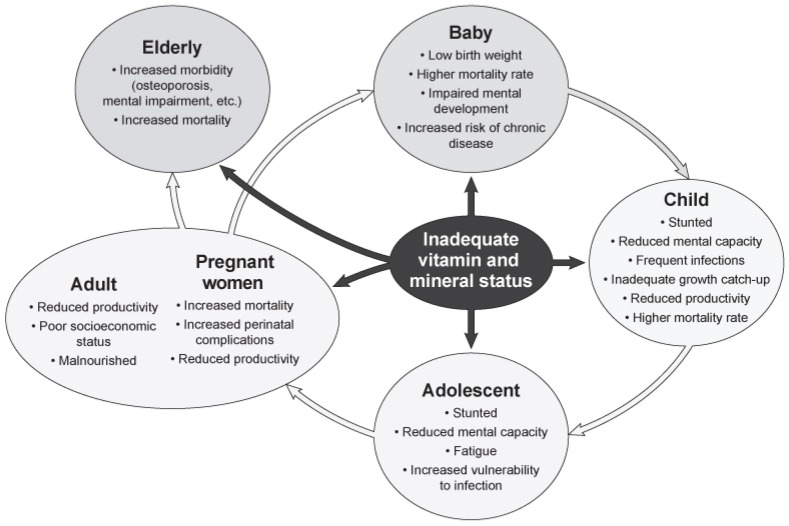
The cycle of micronutrient inadequacies across the life span [7]. Reproduced from Bailey, R.L. et al., with permission from S. Karger AG, Basel.

Undernutrition and micronutrient deficiencies often occur in each part of a vicious intergenerational cycle (Figure 3). Pregnancy and lactation are associated an increased need for specific vitamins and minerals when, particularly in situations of poverty, usual intakes fall short of recommended allowances. These pregnant and lactating women may then have children with suboptimal nutritional status associated with impaired physical and mental development. The poor nutritional background of the infant leads to a trajectory for stunting, increased risk of infection, and/or developmental delays [109]. These children will eventually enter their reproductive years at a nutritional disadvantage so that the cycle will continue. Importantly, adults with these nutritional inadequacies and deficits are likely to have lower work capability, due to their early developmental delays, which are often mediated via a lack of educational attainment [7].

Inadequate vitamin and mineral intake can result from poor dietary patterns associated with low socioeconomic status, barriers to ready access of healthy food, incorrectly constructed programs for weight loss, eating disorders or other emotional and physiological stresses, insufficient nutrition knowledge regarding food shopping, preparation and storage, and untoward lifestyle practices such as smoking [106]. In addition, as aforementioned, increased micronutrient requirements during pregnancy and lactation, older age, substance abuse, and chronic disease can also lead to inadequate vitamin and mineral status [106].

Suboptimal nutrient status in adults is associated with the risk of several chronic diseases, including CVD, osteoporosis, and cancer, suggesting that monitoring, assessment, and intervention are important across all socioeconomic groups in the Middle East [53,110]. Evidence from both observational studies and clinical trials suggest that sufficient intake of vitamins and minerals, often derived from a combination of diet, fortified foods, and supplements, is associated with a reduced risk of inadequate micronutrient intakes, general health promotion, and/or a reduced risk of chronic disease [53,106,110,111,112,113,114]. Nonetheless, in the Middle East, such evidence is absent from long-term prospective cohort studies and from randomized clinical trials examining the efficacy of food patterns and dietary supplementation on reducing the risk for micronutrient deficiency/inadequacy and the risk for non-communicable diseases.

## 5. Food Fortification and Dietary Supplementation in the Middle East

### 5.1. Fortification Practices in the Middle East

Food fortification or enrichment with micronutrients is an attractive, cost-effective solution mandated by government authorities and/or implemented by the food industry to address common inadequacies and deficiencies [115]. Even in countries with food fortification programs, the effectiveness of the initiative must be determined via laboratory testing and/or mobile assessment of micronutrient composition in conjunction with ongoing monitoring of the prevalence and incidence of malnutrition. However, in the Middle East, these programs are often small, outdated, and lacking in quality.

Food fortification has been widely implemented in many countries in the Middle East. The WHO launched an initiative in 1999, supported by UNICEF and the Micronutrient Initiative, which led nearly all countries in the region to begin fortifying wheat flour with iron and folic acid by 2009 [116]. At a Joint WHO/Flour Fortification Initiative harmonization workshop held in 2012, it was decreed that flour fortification with iron and folic acid be made mandatory throughout the Middle Eastern region [116]. In addition, a few countries have considered fortification with vitamin A, B-complex vitamins, vitamin D, and zinc.

The Flour Fortification Initiative revealed that all Middle Eastern countries, with the exception of Lebanon, have invested in mandatory micronutrient fortification of wheat flour for locally manufactured and imported products (Table 2) [58,79,80,81,93,94,95,96,97]. However, despite the availability of folic acid fortification in these countries, NTD still continue to affect 10–33 infants/10,000 births (Table 3) [58,79,80,81,93,94,95,96,97]. By contrast, as per WHO guidelines, the prevalence of anemia has shifted to a “moderate” public health problem between 1992 and 2014 [62], with Egypt, KSA, and Oman having the highest burden in the region, particularly among pre-school children and women of childbearing age (Table 3).

**Figure 4:**
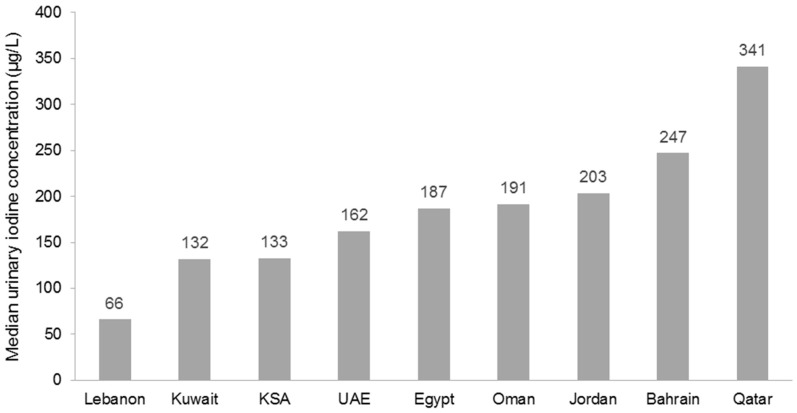
Urinary iodine concentrations in the 2015 Iodine Global Network Scorecard [118].

However, this workshop concluded there was an urgent need for countries in the Middle East to update their national data so as to better inform policy and program development. As an example of such an effort, the Iodine Global Network scorecard for 2015 revealed that most Middle East countries now have adequate iodine status, except in Lebanon where urinary iodine concentration remains inadequate and in Qatar where it appears excessive (Figure 4) [117].

Scarcity of minerals such as iron, iodine, calcium, and zinc often result in several disorders and life-threatening conditions [119]. Of note, double salt fortification with iron and iodine could be a reasonable approach to preventing both iodine and iron deficiencies [119]. Since, stability and bioavailability of both iron and iodine is difficult, salt encapsulation is a novel approach that may provide a physical barrier for undesirable reactions and interactions during storage [119]. Two recent publications rightly pointed out that food fortification is one of the best approaches to alleviating mineral deficiency and impacting overall health of the general population [118,119].

### 5.2. Dietary Supplementation in the Middle East

In addition to food fortification, dietary supplementation provides an individually targeted approach to addressing micronutrient inadequacies and deficiencies. For example, vitamin A supplementation of children <2 years of age, particularly in Asia, represents an established approach to preventing irreversible blindness caused by deficiency during early childhood [115]. Although dietary supplements are available in the Middle East region, information from the scientific literature and government reports are scarce regarding patterns or prevalence of their use by age, sex, education, dietary intake, and/or socioeconomic status.

In the KSA, Seidahmed et al. [90,91] and Safdar et al. [90,91] found the use of folic acid supplements to be extremely low (≤2%) in women of child-bearing age and 10%–60% in pregnant women. Other studies in the KSA by Musaiger et al. [120,121] and Afshin et al. [120,121] revealed that >80% of women aged 18–45 years were unaware of the role played by periconceptional folic acid supplementation. By contrast, in ranges across surveys from the Gulf and Levant region found that 46%–62% of women are aware of the benefits of folic acid and 6%–45% of pregnant women reported taking folic acid supplementation during their first-trimester; the lowest rate of folic acid supplement use was found in Lebanon (6%–14% of women) [122,123,124,125]. In the Feeding Infants and Toddlers Study, Abdulrazzaq et al. evaluated the nutrient adequacy of 1000 infants and toddlers in the UAE and found that, although protein and energy intakes approximated the recommended allowance, iron intake was inadequate (<70% of RDA) in 66.8% of infants 6–11 months old and 100% of toddlers 12–23 months old [126].

Using self-reported data from 270 pregnant Egyptian women, Ibrahim et al. reported that 51.1% of women did not adhere to folic acid and iron supplementation; a pill count assessment revealed non-adherence to be 63.3% [76]. On the other hand, over one-third of women who gave birth during the five-year period prior to the 2008 EDHS, reported taking iron tablets or syrup during the pregnancy preceding their last live birth; a decline in compliance rate from 49% (in 2005) to 33% (in 2008) was observed in the two EDHS reports [127]. Moreover, according to the 2014 EDHS, 72% of mothers who had four or more antenatal care visits were given or bought iron supplements compared with 54% of mothers who had one to three visits, and 26% of mothers who had no antenatal care prior to the last birth [72]. Egypt maintains a vitamin A supplementation program for children beginning at nine months of age; however, compliance is poor with 44% and 49% of children aged 9–11 and 18–27 months receiving this supplement, respectively [127]. The Egyptian Nutrition Landscape Analysis Report of 2012 concluded that current strategies for food fortification and dietary supplementation of vitamin A had been suboptimal and noted that, in conjunction with these data, there was an increase in wasting, stunting, and underweight between 2005 and 2008 [127]; however, this increase may be due to sampling variability, so caution is warranted when interpreting these results. This report also noted that higher micronutrient intake was associated with maternal education, affluence, and area of residence [127].

In a cross-sectional survey of 194 randomly selected medical students from the KSA, Allam et al. found that mineral consumptions were below the recommended daily intake (RDI) for calcium, magnesium, potassium, and zinc (47.0%, 24.5%, 31.3%, and 40.7%, respectively) [128]. Gender comparisons showed that potassium and magnesium intakes were lower among males and calcium intakes were lower among females. The percentage RDI of iron was lower among females, especially from animal sources, than males at 2.2 mg and 3.6 mg, respectively. Intakes of all vitamins were <70% of the RDI in this cohort, with severe deficiencies for vitamin D, vitamin B2, folic acid, and niacin (14.2%, 49.4%, 53.6%, and 67.5%, respectively). Males were also found to have a lower percentage RDI than females for vitamins A, B1, B6, C, and E.

## 6. Benefits and Safety of Dietary Supplementation

### 6.1. Benefits of Dietary Supplements

Systematic reviews have documented the benefits of providing dietary supplements of iron, vitamin D, vitamin A, zinc, calcium, and iodized salt in children and adolescents [74,129], women [45,74,130,131], and the elderly [53,110,132,133].

#### 6.1.1. Children and Adolescents

A Cochrane review of 33 randomized controlled trials (RCTs) found that intermittent iron supplementation in 13,114 children aged <2 years (~49% female) reduced the risk of anemia and IDA by 49% and 76%, respectively [74]. Similarly, the same Cochrane review of 43 RCTs reported that vitamin A supplementation in 215,633 children aged <5 years reduced the risk of all-cause mortality by 24%, diarrhea-related mortality by 28%, and the incidence of diarrhea by 15% and measles by 50% [74]. In children at risk of zinc deficiency, preventive supplementation has been shown in 18 RCTs to reduce the incidence of diarrhea by 13% and pneumonia by 19%; a non-significant reduction of 9% in all-cause mortality was reported, although a sub-group analysis of children aged 1–5 years showed an 18% reduction in the risk of all-cause mortality [74]. Studies of preventive zinc supplementation have reported that increasing zinc intake in at-risk populations increases children’s weight and linear growth, thereby reducing the prevalence of stunting [129].

#### 6.1.2. Women

Data suggest the current total deaths in children <5 years of age can be reduced by 15% if maternal populations can access ten evidence-based nutrition interventions at 90% coverage [74]. A systematic review of five RCTs including 6105 women showed that periconceptional folic acid supplementation reduces the risk of NTD by 72% and risk of recurrence by 68% compared with no intervention, placebo, or micronutrient intake without folic acid [74]. In a systematic review of 31 RCTs testing folic acid supplementation in 17,771 women during pregnancy, the incidence of megaloblastic anemia was reduced by 79% [74]. The Cochrane review of 21 RCTs of intermittent iron supplementation in 10,258 non-pregnant women of reproductive age found that the risk of anemia was reduced by 27%; a similar effectiveness of daily iron supplementation during pregnancy was found in 43 RCTs involving 27,402 women showing risk reductions of 70% in anemia at term, 67% in IDA, and 19% in the incidence of low birthweight [74].

Multiple micronutrient deficiencies often co-exist and can lead to potential adverse maternal outcomes [74]. A Cochrane review of 21 RCTs involving 75,785 women taking multiple micronutrient supplementation during pregnancy reported an 11%–13% reduction in low birthweight and small-for-gestational age births [74]. Importantly, no adverse effects on maternal or neonatal mortality were recorded [74].

In Cochrane analyses, calcium supplementation during pregnancy in women at risk of low calcium intake was found to reduce the incidence of gestational hypertension by 35% (11 RCTs, *n* = 14,946), pre-eclampsia by 55% (12 RCTs, *n* = 15,206), and preterm births by 24% (10 RCTs, *n* = 15,141) [74]. A more recent pooled analysis of 15 RCTs, showed that calcium supplementation during pregnancy reduced the risk of pre-eclampsia by 52% in those at risk of low calcium intake [74]. The use of iodized salt has been shown to be an effective means to improving iodine status; however, no conclusions could be drawn from this review regarding physical and mental development in children and overall mortality; thus, there remains a need for further investigation regarding iodine fortification and supplementation [74].

A RCT comparing the effect of UNICEF/WHO/United Nations University multiple micronutrient supplement for pregnant and lactating women (UNIMMAP), with the usual iron and folic acid supplement (IFA), on survival, growth, and morbidity during infancy (*n* = 1169 livebirths eligible for growth monitoring), found that prenatal multivitamin use resulted in a 27% reduction in stunting rate during the first year of life [131]. Children whose mothers had received this multiple micronutrient supplement also had significantly greater z scores for length-for-age, weight-for-age, thoracic circumference, mid-upper arm circumference, and head circumference-for-age [131].

A Cochrane review of 15 RCTs involving 2833 women concluded that supplementing pregnant women with vitamin D in a single or continued dose regimen increases serum 25(OH)D at term and may reduce the risk of pre-eclampsia, low birthweight, and preterm birth [130]. Prolonged breastfeeding without vitamin D supplementation and low dietary calcium intake are also recognized risk factors for rickets and hypovitaminosis D in children [45]. However, further investigations into the range of benefits of vitamin D supplementation during pregnancy are still warranted [130].

#### 6.1.3. Older Adults

In a systematic review of 37 studies including >20,000 subjects, ter Borg et al. [132] concluded that micronutrient supplementation of community-dwelling older adults is likely to prevent fractures, increase bone density, preserve muscle mass and performance, and reverse deficiencies and their potential consequences. Vitamin D supplementation has been shown to decrease bone turnover, increase bone mineral density, with measurable decreases in parathyroid hormone [53]. Supplementation of vitamin D and calcium has been shown to decrease bone loss and fracture rates in the elderly [53,110].

When considering meta-analyses of prospective studies, McNulty et al. concluded that increasing the status of vitamin B6, vitamin B12, and folic acid sufficiently to lower total plasma homocysteine by 3 mmol/L was associated with a reduction in the risk of CVD by 11%–16% and of stroke by 19%–24% [133]. A meta-analysis of RCTs found that folic acid supplementation alone reduced the overall risk of stroke by 18%, and by 29% in RCTs with a treatment duration of >36 months, and by 25% in individuals without a history of stroke [133]. These findings are consistent with data reported from observational studies in the USA and Canada that showed a temporal decline in stroke-related mortality with the introduction of folic acid fortification.

To date, no large-scale, long-term prospective cohort studies or RCTs of the potential benefits of dietary supplementation on chronic disease outcomes have been undertaken in the Middle East region

### 6.2. Safety of Dietary Supplements

Risk assessment models used to derive tolerable upper intake levels, safe upper levels, or guidance levels for vitamins and minerals have been established by advisory or regulatory authorities in Australia, Europe, New Zealand, United Kingdom, and the USA [134]. As it is unethical and infeasible to conduct clinical trials targeted at adverse outcomes, these values are based on harm observed in animal model studies and/or available case reports of clinical evidence of toxicity. Nonetheless, the setting of these levels provides a framework within which a healthcare provider or consumer can make informed decisions about intake, with confidence that no harm should ensue.

The possibility of harm from consuming supplemental vitamin and mineral doses that exceed the recommended amount depends on the supplement type, amount consumed, and the nature of the dose–response relationship of the micronutrient [135].

Some observational studies and meta-analyses of RCTs of nutrient supplements, often at supra-dietary doses, suggest that their use may be associated with increases in total mortality [136,137,138]. By contrast, a meta-analysis of 21 RCTs including 91,074 subjects with a mean age of 62 years, conducted by Macpherson et al., found that across all studies, multivitamin–multimineral supplementation had no effect on cancer or vascular mortality, but resulted in a slight decrease in all-cause mortality following an average duration of 43 months supplementation [139]. Minor or short-term untoward effects of some dietary supplements, including gastrointestinal symptoms, cited in cases or adverse event reports, are apparently readily reversible upon withdrawal of the supplement [135]. Interactions between dietary supplements and drug therapy or polypharmacy regimens may result in untoward consequences associated with alteration of the efficacy of either treatment [135,140]. Interestingly, no limitations are placed on the use of fortified foods because no relevant safety data are available.

## 7. Impact of Malnutrition on Healthcare Costs

Data on the healthcare cost of malnutrition from the Middle East region are lacking. However, a review of international literature highlights the high cost of micronutrient deficiencies on economic productivity, school achievement, gross domestic product (GDP), and degree of development status [141,142].

The global cost-effectiveness analysis of salt iodization and flour fortification as well as vitamin A and zinc supplementation has been well established [143]. The Copenhagen Consensus, an international collaboration of economists, has provided cost–benefit analyses that estimate the annual loss in GDP associated with inadequate nutrition is up to 12% in poor countries [141]. Therefore, with the low cost of interventions and the unmatched benefit-cost ratio of micronutrient programming, supplementation and fortification initiatives should be integrated into ongoing health services and existing food production methods [143].

Importantly, micronutrients are inexpensive, with low cost supplements and fortificants available via an array of commercial channels [143]. Although neither food fortification nor dietary supplementation can address the primary causes of poor micronutrient status, they do provide the quickest amelioration of micronutrient status for individuals or targeted populations [144]. Fortification programs can focus on widely consumed staple foods within a country, while dietary supplementation can provide micronutrients in effective doses and delivery forms targeted to specific population groups. Regrettably, analyses of fortification practices in some regions, including the Middle East, reveal that the quality of these fortified foods is low. Generally, there are limited data on how compliant fortification programs are, so there is a need to ensure governments and programs have the capacity and tools required to effectively track and take action with the food industry regarding food quality issues.

## 8. Proposed Strategies to Address Nutrition and Healthcare Challenges

The 2010–2019 strategy developed by the WHO Regional Office for the Eastern Mediterranean emphasizes that undernutrition, and its associated impact on the burden of disease in the Middle East, must be addressed via multiple, concurrent approaches that build institutional capacity by encouraging public, private, and nongovernmental organizations to affect the nutritional content of processed foods and to educate consumers to make healthy food choices [2]. However, despite many existing and published national nutrition policies and action plans promulgated by Middle Eastern countries, most have not been fully implemented and lack a clear political or strategic path forward. Therefore, a main goal of policy should be to encourage Middle Eastern countries to reposition nutrition and the prevention of malnutrition as central to their development; this can be achieved by implementing the strategies, summarized below, to improve the nutritional status of the population throughout their life time. In this regard, both short- and long-term goals have been identified.

The principal short-term goals are the need for greater nutrition education and government-led initiatives to reduce malnutrition. There is a need for educational programs targeting populations with the greatest vulnerability for malnutrition, including women of child-bearing age, pregnant women, children (especially during their “first 1000 days”), and the elderly. Programs for healthcare professionals are required on the identification of nutritional risk, inadequacy, and deficiency and, accordingly, effective recommendations should be established for prevention and treatment. From a public health perspective, efforts are required to create and strengthen nationwide nutrition surveillance systems and to improve the use and reporting of relevant indicators of malnutrition and food security. Results from this monitoring should be quickly translated into food, fortification, and supplement initiatives that address micronutrient shortfalls and associated deficiency syndromes.

The major long-term goals include the implementation of effective programs encompassing new research and evidence-based government policies. Despite marked economic growth in several Middle East countries, few resources have been directed to monitoring primary health and nutrition information, and to establishing related national healthcare databases. The majority of available data on nutritional status in the Middle East region have been generated from small-scale observational studies that often lack results that are generalizable to the larger population. Although RCTs of primary and secondary prevention of malnutrition have been conducted outside of the Middle East, only local and regional studies can determine the advantages and limitations associated with translating and implementing culturally appropriate approaches within this region.

Lastly, public–private partnerships are key to achieving and sustaining both short- and long-term nutrition goals. As noted at the Joint WHO/Flour Fortification Initiative harmonization workshop in 2012, it is important that nutrition interventions are not conducted in isolation, but integrated into policies and programs in different sectors [116]. Although education and food fortification are integral to the solutions for addressing micronutrient malnutrition, using dietary supplementation to target specific populations should be encouraged via training relevant healthcare providers and also by empowering consumers through education to take action for themselves and their families [116]. Programs to increase supplement use should be supported with the development of a database for monitoring supplement use and its impact on combating micronutrient diseases.

## 9. Conclusions

The Global Progress Report of the Micronutrient Initiative and UNICEF quotes the World Bank, stating, “The control of vitamin and mineral deficiencies is one of the most extraordinary development-related scientific advances of recent years. Probably no other technology available today offers as large an opportunity to improve lives and accelerate development at such low cost and in such a short time” [142]. These advances point beyond the classical goal of preventing obvious vitamin and mineral deficiencies in the Middle East, and to the recognition that less severe (but nonetheless inadequate) micronutrient status often presents with no clinical symptoms but can interfere with intellectual development and lead to ill health. This may, in turn, contribute to a vicious intergenerational cycle of impaired health and function. Thus, this review highlights not only the prevalence of overt deficiency syndromes, but also the “mild” or “moderate” levels of micronutrient inadequacy common in the Middle East region. This situation demonstrates the need for physicians to reach out not only to the most vulnerable groups, but also to whole populations, especially those undergoing nutrition transition. By taking advantage of multiple approaches to encourage healthy dietary patterns as well as promoting micronutrient sufficiency via food fortification and dietary supplementation, improvements in the Middle East region will be seen in the future.

## Figures and Tables

**Table 1:** Country estimates of the prevalence of night blindness and vitamin A deficiency in preschool children and pregnant women (1995–2005) ^a^.

Country	Preschool Children		Pregnant Women	
	Prevalence of Night Blindness	Prevalence of Vitamin A Deficiency (<0.7 µmol/L)	Prevalence of Night Blindness	Prevalence of Vitamin A Deficiency (<0.7 µmol/L)
Lebanon	0.6%	2%	3.7%	3%
Jordan	0.6%	4%	4.4%	7%
Egypt	0.1%	9%	9.4% ^b^	10.2% ^b^
KSA	0.4%	12%	5.1%	32%
Oman	0.4 %	1 %	%	3 %

^a^ Table derived from reference [104]; except ^b^ taken from [105].

**Table 2:** Nutrients added for wheat flour fortification (parts per million) ^a^.

Country	Iron	Zinc	Folic Acid	B12	Niacin	Riboflavin	Thiamine	Vitamin A	Percent Flour Fortified
Lebanon	No fortification								
Jordan	32.25 (ferrous sulfate)	20	1	0.007	35	3.6	3.575	1.5	100% (mandatory)
Egypt	30 (ferrous sulfate)	-	1.5	-	-	-	-	-	Unavailable
KSA	36.3 (type unknown)	-	1.5	-	52.9	3.96	6.38	-	100% (mandatory)
UAE	60 (electrolyte)	-	1.5	-	-	-	-	-	90% (mandatory)
Oman	60 (electrolyte)	-	1.5–2.0	-	-	-	-	-	89% (mandatory)
Bahrain	60 (electrolyte)	-	1.5	-	-	-	-	-	90% (mandatory)
Qatar	60 (electrolyte)	-	1.5	-	-	-	-	-	90% (voluntary)
Kuwait	60 (electrolyte)	-	1.5	-	52.91	3.96	6.38	1.5	100% (mandatory)

^a^ Table derived from references [74,83,84,85,86,87,88,89,90].

**Table 3:** Neural tube defects and anemia status in selected countries of the Middle East ^a,b^.

Country	Neural Tube Defects per 10,000 births	Percent Anemia in Non-Pregnant Women of Reproductive Age	Percent Anemia in Pre-School Children
Lebanon	18.0	28	24
Jordan	33.0	29	31
Egypt	10	35	45
KSA	12.0	40	39
UAE	12.0	26	29
Oman	12	35	41
Bahrain	11.8	38	32
Qatar	12	28	26
Kuwait	12	22	26

^a^ Table derived from references [74,83,84,85,86,87,88,89,90]; ^b^ Neural tube defects and anemia values generated post fortification with folic acid.

## Abbreviations

The following abbreviations are used in this manuscript:

CMD	cardiometabolic disease
CVD	cardiovascular disease
EDHS	Egyptian Demographic and Health Survey
FAO	Food and Agriculture Organization of the United Nations
GDP	Gross Domestic Product
Hb	hemoglobin
IDA	iron deficiency anemia
IU	international units
KSA	Kingdom of Saudi Arabia
NTD	neural tube defect
RCT	randomized controlled trial
UAE	United Arab Emirates
UNICEF	United Nations Children’s Fund
USD	United States dollar
WHO	World Health Organization
